# Oropharyngeal Airway Three-dimensional Changes after Treatment with Myobrace in Class II Retrognathic Children

**Published:** 2017-02

**Authors:** Eun-Suk AHN, Ah-Hyeon KIM, Youn-Soo SHIM, So-Youn AN

**Affiliations:** 1. Dept. of Dental Hygiene, Kyungbok University, Seoul, Republic of Korea; 2. Dental Spa Dental Clinic, Daejeon, Republic of Korea; 3. Dept. of Dental Hygiene, College of Health Sciences, Sunmoon University, Cheonan, Republic of Korea; 4. Oral Health Research Institute, Korean Academy of Systematic Stomatology, Seoul, Republic of Korea; 5. Dept. of Pediatric Dentistry, College of Dentistry, Wonkwang University, Iksan, Republic of Korea

## Dear Editor-in-Chief

Several scholars pointed out the relationship between the function of muscle and teeth (1). The effects of breathing on facial growth and occlusion of the bite have been identified in various papers (2, 3). A breathing disorder occurs after upper airway blockage during sleep, it has not only nighttime symptoms such as snoring or apnea but also daytime symptoms such as behavioral disorder, excessive daytime sleepiness, concentration issue, memory, and cognitive function.

Sleep is a physiological phenomenon essential for performing basic human functions and is important for normal growth and development, and for maintaining emotional health and immunity. Among various sleep disorders, Obstructive Sleep Apnea Syndrome (OSAS) is a common medical problem for children (4). Among the sleep disorders, obstructive sleep apnea has been reported in Korean children and adolescents based on polysomnography, relative to other sleep disorders. Sleep-disordered breathing can be prevented myofunctional therapy, the neuromuscular toning and re-education of oral, facial, and pharyngeal muscles (lips, tongue, cheeks, face, and throat) through a series of activities which helps normalize the developing or developed, head and neck structures and function, with orthodontic treatment (5,6). A recent research literature review shows myofunctional therapy for those whose tongues block their airway during sleep can reduce these choking events by approximately 50% in adults and 62% in children and those lowest blood oxygen saturations, snoring, and sleepiness outcomes improve in adults (7).

We now have the evidence-based studies to validate the success of treating patients collaboratively with a variety of disorders (e.g., orthodontic disorders, sleep apnea, orofacial pain) but, until recently, there were not enough studies using oral appliance and myofunctional therapy of Korean children and adolescents. Since cephalometric radiography was introduced by Broadbent to evaluate the craniofacial morphology, as recently reported (8), to evaluate the size of the airways and tongue in patients with sleep apnea, it is widely used, but, not be evaluated in three dimensions.

**Fig. 1:**
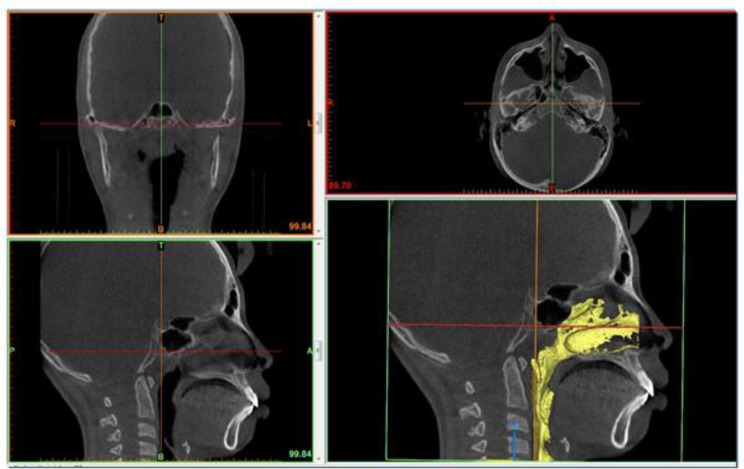
Clinical radiograph of case using Myobrace (T4A^TM^): Before treatment CBCT

**Fig. 2:**
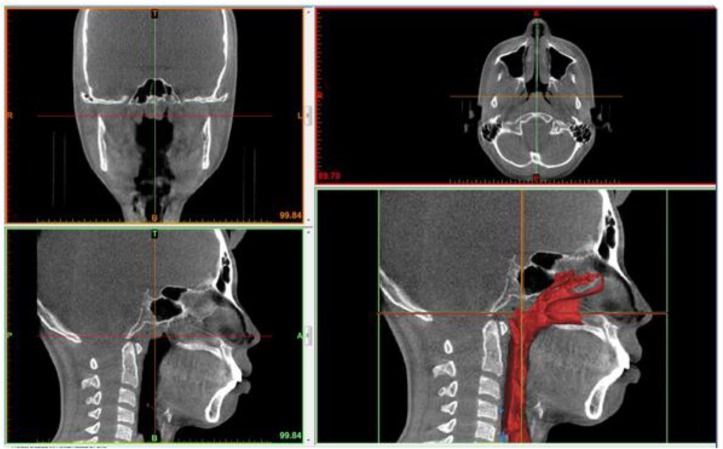
Clinical radiograph of case using Myobrace (T4A^TM^): after treatment CBCT

**Fig. 3:**
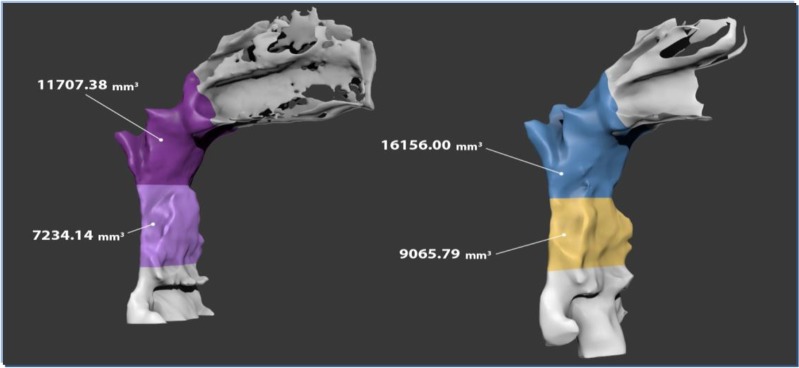
Comparison of three-dimensional measurement of before and after treatment

Cone-beam computed tomography (CBCT) is a useful nondestructive method for 3D reconstruction that can be used to visualize and measure the morphological structures of hard and soft tissue on face. 3D models reconstructed from CBCT and tomographic images can display the precise anatomical structure of Oropharyngeal space. In addition, CBCT is useful for establishing real-time clinical diagnoses of patients and treatment plans (9). Programs that accurately measure complex structures of 3D models mathematically are available, but no studies have attempted to measure the Oropharyngeal space area using CBCT technology. So, our study was conducted to evaluate three dimensional changes after Myobrace (Myofunctional Research Co, Australia) appliance treatment for the children with OSAS symptoms (Fig. 1, 2). Our study confirmed the increase of oropharyneal airway dimension using Myobrace (Fig. 3). We excluded the sleep symptoms of the participating patients.

Although the majority of childhood obstructive sleep apnea is due to hypertrophy of adnexaadenoid, there is no correlation between the magnitude of hypertrophy and the severity of obstructive sleep apnea syndrome. So far, polysomnography that it is necessary to confirm the sleep apnea test if sleep apnea is suspected especially in children, is the most important diagnosis for obstructive sleep apnea. However, the cost of testing is high, and labor and time are required to inspect and read. Especially, when performing polysomnography, it is necessary to attach several electrodes. For this reason, this study failed to provide an objective validation of the improvement of symptoms of sleep disorders through polysomnography before and after myobrace treatment. Additional studies are necessary for objective improvements of symptoms of sleep disorders using polysomnography after applying Myobrace to the patients diagnosed with OSA.

Since we obtained airway expansion using Myobrace, we suggest it is used for myofunctional orthodontic treatment of childhood OSAS patients.
